# Retracted: Immunomodulation in Human Dendritic Cells Leads to Induction of Interferon-Gamma Production by *Leishmania donovani* Derived KMP-11 Antigen via Activation of NF-*κ*B in Indian Kala-Azar Patients

**DOI:** 10.1155/2022/9854346

**Published:** 2022-12-07

**Authors:** Biomed Research International

*Biomed Research International* has retracted the article titled “Immunomodulation in Human Dendritic Cells Leads to Induction of Interferon-Gamma Production by Leishmania Donovani Derived Kmp-11 Antigen via Activation of Nf-*κ*b in Indian Kala-Azar Patients,” [1], due to concerns raised regarding apparent duplications of features between and within figures within the manuscript [2].

Specifically, the same formation of data points within the dot plot of image Figure 2(b) B1 appear to be present in Figure 2(e). There also appears to be a further duplication of data points within Figure 2(e). Finally, data points within Figure 3(b) appear to be duplicated between the Macrophage KMP-11 sti panel and the Dendritic cells KMP-11 sti panel.

The authors were unable to provide the raw data when contacted regarding these concerns. Hence this article is being retracted due to concerns regarding the validity of the results and conclusions.
